# Should Medical Practitioners Dispense?

**Published:** 1910-07-02

**Authors:** 


### SPECIAL ARTICLES.

### SHOULD MEDICAL PRACTITIONERS DISPENSE?

[*The question which heads this article is one that is arousing considerable attention at the present moment, and in the circumstances the opinions of practical men, looking at the subject from various standpoints, are of considerable interest. We shall be glad to receive further communications from practitioners upon the question.—Ed.* The Hospital.]

### III.—PRESCRIBER AND DISPENSER.

A GENERAL PRACTITIONER'S VIEW.

The subject of the dispensing doctor and the prescribing chemist is a very old one, but it seems evergreen and as little advanced towards solution as ever it was. The views of a hospital physician—presumably, therefore, a strict consultant—and of a pharmaceutical chemist have already been given (The Hospital, June 25, 1910, pp. 379, 380), and it is on this account that an ordinary general practitioner now ventures to put forward that side of the question of which he sees the most.

Let it be premised that there is a great deal of reason in both the doctor's objection to counter prescribing and the chemist's to surgery dispensing. Let it be admitted, though most medical men contest it when discussing this topic, that the medical student of to-day does *not* receive during his studies that training in dispensing, in pharmacy, and in materia medica which is theoretically desirable. This admission is made because it is, in the author's belief, a true statement; but nominally the training is adequate, according to the theory of the curriculum. However, it may safely be said that in these subjects the knowledge of the chemist is superior—nay, very greatly superior—to that of the doctor. *Per contra*, it is just as patent to everyone who is conversant with the routine of medical practice that what even the most highly intelligent and best trained chemist knows about disease, diagnosis, and treatment is so little as is just enough to make that knowledge highly dangerous to the community at large.

When all is said and done, it comes to this: that medical men in the country and in the less affluent districts of towns dispense their own prescriptions, not because they like it (ninety-nine out of a hundred hate it), but because they must do so in order to live; while chemists, who persistently refuse to recommend and sell remedies for anything their customers choose to fancy is the matter with them, are equally likely to give offence and to lose their livelihood. Which of the two classes does the more in poaching on the preserves of the other it is not easy to say. The blame of the muddle really lies with the public, which steadily refuses to recognise that in the pursuit of health it is no economy to employ either a good doctor who is but a moderate dispenser, or a good chemist who knows little of medicine and surgery. As one of the medical profession I should like to argue that of the two the doctor who dispenses is less likely to do harm and more likely to do good, than the chemist who prescribes; but that may be merely prejudice on my part.

It remains to scrutinise closely one or two of the paragraphs in the "Pharmaceutical Chemist's" article. This writer says: "If . . . it is intended to convey the impression that chemists as a body make a practice of affecting to diagnose diseases and of treating each patient as a special case, it is quite wrong to say that chemists prescribe." Now this is quite correct as it stands; no doubt chemists are few and far between who pretend to any clinical experience or rely on physical signs. But at the same time chemists do all day long dispense medicines from their own prescriptions for patients and their friends.

It is undeniable, for it is an everyday and familiar thing in general practice, that chemists will, and do, send out medicines for people they have never even seen; the "fever mixture" is a common example, and no better instance could possibly be chosen of the danger and scandal of counter-prescribing. The author could, if he would, give the name of an examiner (or ex-examiner) to the Pharmaceutical Society who does this to a considerable extent. This chemist is a highly cultured and intelligent expert in his own department, and there are probably few in his craft who deserve and command higher respect. Yet he thinks nothing of sending out a (doubtless harmless) febrifuge mixture when relatives come to his shop with a tale of "feverishness" in someone at home. This pharmacist always refers at once to a doctor (frequently to the present writer) anyone whom he suspects to be suffering from any serious illness; but, acute and experienced as he is, he passes on cases that are trivial, while now and then he undoubtedly jeopardises human life by not appreciating his own limitations and wasting valuable time instead of advising consultation with a doctor. It is all very well for apologists such as the "Pharmaceutical Chemist" to tell us that chemists merely stock simple remedies for minor ailments and sell them without pretending that they are anything but "stock" remedies. The public most certainly does not understand that; it goes to the chemist to be treated for its disease, and whether it makes its own diagnosis or asks the chemist to make his from its verbose and unsifted "history," the potentiality of evil that ensues is equally great. It is just because no chemist can discriminate between what is a minor ailment and what is the early stage of a mortal one that he has no business to try or to allow the patient to try. Admittedly the foolish and ignorant importunity of the latter causes much of the mischief; but the chemist must certainly bear a share in the blame of fostering it.

